# Effect of Perineural or Intravenous Betamethasone on Femoral Nerve Block Outcomes in Knee Arthroplasty: A Randomized, Controlled Study

**DOI:** 10.1111/os.14018

**Published:** 2024-02-21

**Authors:** Binglong Li, Xiaomei Yang, Fang Liu, Huang Huang, Baoqing Zhang, Xuezhou Li, Qunshan Lu, Peilai Liu, Lixia Fan

**Affiliations:** ^1^ Department of Orthopedics Qilu Hospital of Shandong University Jinan China; ^2^ Shandong University Cheeloo College of Medicine Jinan China; ^3^ Department of Anaesthesiology, Qilu Hospital Shandong University Jinan China; ^4^ Department of Cardiology, the Key Laboratory of Cardiovascular Remodeling and Function Research, Chinese Ministry of Education, Chinese National Health Commission and Chinese Academy of Medical Sciences, the State and Shandong Province Joint Key Laboratory of Translational Cardiovascular Medicine Qilu Hospital of Shandong University Jinan China; ^5^ Institute for In Vitro Sciences Gaithersburg MD USA

**Keywords:** Analgesia, Arthroplasty, Betamethasone, Femoral nerve block

## Abstract

**Objectives:**

Despite the use of multimodal analgesia, patients undergoing knee arthroplasty still encounter residual moderate pain. The addition of betamethasone to local anesthetic has been shown to improve postoperative pain. However, it remains uncertain whether the positive effects of perineural or intravenous administration of betamethasone on analgesia outcomes lead to better early mobility and postoperative recovery.

**Methods:**

Between June 2022 and February 2023, a total of 159 patients who were undergoing knee arthroplasty were included in this study. These patients were allocated randomly into three groups: (i) the NS group, received ropivacaine 0.375% and intravenous 3mL 0.9% normal saline; (ii) the PNB group, received ropivacaine 0.375% plus perineural betamethasone (12mg) 3mL and intravenous 3mL 0.9% normal saline; and (iii) the IVB group, received ropivacaine 0.375% and intravenous betamethasone (12mg) 3mL.

**Results:**

Both perineural and intravenous administration of betamethasone led to improved median (IQR) numeric rating scale (NRS) scores on the 6‐meter walk test, with a score of 1.0 (1.0–2.0) for both groups, compared with 2.0 (1.0–2.0) for the NS group (*p* = 0.003). Compared to the NS group, both the PNB and IVB groups showed significant reductions in NRS scores at 24 and 36 h after surgery, along with a significant increase in ROM at 24, 36, and 48 h post‐operation. Additionally, it exhibited lower levels of cytokine IL‐1β and TNF‐α in fluid samples, as well as lower level of HS‐CRP in blood samples in the PNB and IVB groups compared to the NS group.

**Conclusion:**

The administration of perineural and intravenous betamethasone demonstrated an enhanced analgesic effect following knee arthroplasty. Furthermore, it was associated with reduced levels of IL‐1β, TNF‐α, and HS‐CRP, as well as enhanced knee ROM, which is conducive to early ambulation and postoperative rehabilitation after knee arthroplasty.

## Graphical abstract

A: violin plot of 6‐meter walk test NRS score at 24 h after surgery. The purple solid line represents median and the black dotted line represents interquartile range (IQR). B: the concentration of HS‐CRP in blood at preoperative and postoperative. C: the concentration of IL‐1β in joint fluid at 24 h after surgery. D: the concentration of TNF‐α in joint fluid at 24 h after surgery. *Statistically significant difference, *p* < 0.05; **Statistically significant difference, *p* < 0.001.

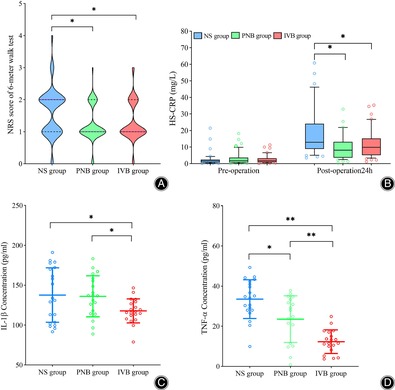

## Introduction

While knee arthroplasty effectively alleviates pain in patients with end‐stage knee osteoarthritis, significant postoperative pain is expected and can hinder mobilization and recovery. During the procedure, severe postoperative pain is often associated with soft tissue exposure and extensive osteotomy. As part of a multimodal analgesia approach, ultrasound (US)‐guided femoral nerve block (FNB) has been shown to reduce morphine consumption by an average of 20 mg within 24 h and 38 mg within 48 h, compared to patient‐controlled analgesia (PCA). Additionally, FNB demonstrated lower pain levels, with reductions of 1.1 and 1.8 in pain scores at 24 h at rest and during activity, respectively.^1^, ^2^, ^3^ Ropivacaine is commonly employed as a local anesthetic for nerve blocks, offering sensory and motor block durations ranging from 24 to 28 h and 12 to 20 h, respectively.^4^, ^5^ Its low lipid solubility makes it less likely to penetrate the thicker myelin sheaths of motor fibers, resulting in a separation of sensory and motor blockade. This phenomenon is concentration‐dependent, with better sensory blockade and slight motor blockade observed at a lower concentration.^6^

Early postoperative ambulation shortens hospital stays and prevents the risk of complications such as knee stiffness, impaired lung function, muscle atrophy, and venous thrombosis. Nevertheless, once the analgesic effect of the nerve block wears off, patients frequently experience moderate pain.^7^ Pain catastrophizing remains an important concern, particularly in the early stage of recovery after knee arthroplasty, which inhibits early mobility and postoperative recovery. Therefore, it is imperative to optimize the components of the anesthetic protocol to minimize pain and facilitate a swifter postoperative recovery.

To prolong the duration of FNB, studies have explored the use of adjuvants such as dexmedetomidine, clonidine, ketamine, and glucocorticoids which have been added to local anesthetics.^8^, ^9^, ^10^, ^11^, ^12^ Among these, long‐acting glucocorticoids have shown exciting results in extending the duration of FNB. When combined with the local anesthetic ropivacaine, dexamethasone prolongs the duration of analgesia after interscalene brachial plexus blocks, sciatic nerve blocks, and popliteal nerve blocks.^13^, ^14^, ^15^, ^16^ In this study, betamethasone, a long‐acting corticosteroid and stereoisomer of dexamethasone, was used instead of the commonly employed dexamethasone. Betamethasone has been utilized clinically for peripheral nerve injections in patients with chronic pain.^17^, ^18^ Steroids can be divided into particulate and non‐particulate based on aggregation characteristics and solubility. While steroid particles are thought to act as a local reservoir, gradually breaking down and releasing the steroid for an extended effect, betamethasone sodium phosphate and dexamethasone sodium phosphate are pure liquids with no discernible particles.^19^, ^20^ However, in the solution of ropivacaine, betamethasone exists partially in particulate form.

The objective of this study is: (i) to assess whether the administration of perineural or intravenous betamethasone can enhance early mobilization and facilitate postoperative recovery; (ii) to determine whether an optimal protocol can be established to facilitate early mobilization in the context of enhanced recovery after surgery (ERAS); and (iii) to evaluate the safety of the utilization of perineural or intravenous betamethasone.

## Methods

### Ethics Approval and Registration

This study was approved by the Ethics Committee on Scientific Research of Shandong University Qilu Hospital (KYLL‐202206‐018‐1) and registered in the Chinese Clinical Trial Registry (Registration number: ChiCTR2200056974).Informed consent was obtained from all individual participants included in the study.

### Study Population

The study recruited patients between 40 and 80 years, with a body mass index (BMI) ranging from 18 to 40 kg/m,^2^ and an American Society of Anesthesiologists physical status of I‐III, who were scheduled to undergo unilateral knee arthroplasty. The inclusion criteria are listed in Supplementary Table S1. Exclusions are applied to revision knee arthroplasty, rheumatoid arthritis, gouty arthritis, regional nerve block disorders (such as skin ulcers or infections, coagulation disorders), contraindications to general anesthesia (such as severe cardiac insufficiency, severe chronic obstructive pulmonary disease), contraindications to betamethasone based on product instructions (systemic fungal infection, severe osteoporosis, epilepsy), and allergy to the study drug. Before the surgical procedure, the patients provided informed consent by signing the necessary documentation.

### Randomization and Blinding

The patients were randomly divided into three groups: normal saline (NS), perineural betamethasone (PNB), and intravenous betamethasone (IVB) at a ratio of 1:1:1 by computer‐generated random sequence. The results of randomization were numbered sequentially and sealed in opaque envelopes kept by research assistants. On the day of surgery, the opaque envelopes were handed sequentially to anesthesia assistants, then they dispensed medications according to group assignment. The anesthesia assistant was aware of the patient groupings but was not further involved in the study. Patients, anesthesiologists, physicians, and statisticians were blinded.

### Perioperative Management

In the NS group, the FNB injectate mixture consisted of 10mL of 0.75% ropivacaine (75mg/10mL) plus 10mL of 0.9% normal saline, and 3mL of normal saline was injected intravenously 1 h before surgery. In the PNB group, the FNB injectate mixture consisted of 0.75% ropivacaine (75mg/10mL) 10mL, 0.9% normal saline 7mL, and betamethasone sodium phosphate injection (12mg) 3mL (Suicheng Pharmaceutical, Henan, China). In addition, 3mL of normal saline was administrated intravenously 1 h before the surgery. In the IVB group, the FNB injectate mixture consisted of 0.75% ropivacaine (75mg/10mL) 10mL plus 10 mL of 0.9% normal saline. One hour before surgery, an intravenous injection of 3mL betamethasone sodium phosphate injection (12mg) was administered.

A single FNB was implemented by a 50 mm short‐bevel No. 22 nerve stimulation needle (001156–74, PAJUNK, Giesingen, Germany). General anesthesia was then induced with intravenous midazolam (0.02–0.05 mg/kg), etomidate (0.2 mg/kg), sufentanil (0.2 ug/kg), and rocuronium (0.6 mg/kg). After induction of anesthesia, a laryngeal mask of appropriate size was placed, and sevoflurane was used to maintain anesthesia. During the operation, when the blood pressure increased by more than 20% of the preoperative baseline value, an additional appropriate amount of sufentanil was given.

Ten patients in each group were randomly selected to extract the joint fluid with a sterile empty needle before the incision of the joint capsule during operation, and the concentrations of inflammatory factors such as TNF‐α and IL‐1β from joint fluid were detected by enzyme‐linked immunosorbent assay (Elisa). The level of HS‐CRP was assessed from blood samples collected from all patients both 24 h before and 24 h after the surgical procedure.

All patients were administered a standardized treatment and multimodal analgesia. Each patient received 1.5 g of tranexamic acid intravenously and 1.5 g of local joint cavity injection. During the surgery, only fentanyl and its analogs were used for intraoperative medication. Local infiltration analgesia was performed by the surgeon with 100 mg of ropivacaine (100mg 10ml), 7 mg of compound betamethasone injection, and 80mg of parecoxib sodium for injection. Postoperatively, patients received a multimodal analgesia protocol, including oral celecoxib 200 mg every 12 h and oral administration of Baclofen tablets at 5mg three times a day. Patients received oral oxycodone 5mg every 6 h or intravenous morphine 2.5‐5mg if unable to tolerate oral medication as needed for breakthrough pain. The use of any additional rescue medications and patient‐controlled analgesia (PCA) was prohibited.

### Outcomes

The primary outcome of the study was the numeric rating scale (NRS) score for a 6‐meter walk, assessed 24 h after surgery. A score of 0 indicated no pain, while a score of 10 indicated the most severe pain imaginable. The discrepancies in NRS scores were also evaluated among the groups to determine their credentials to meet the minimum clinically important difference (MCID) criteria. According to Myles *et al*., analgesic interventions that provided a change of 10 for the 100 (a change of 1 for the 10) pain scores indicated a clinically important improvement after surgery.^21^ Secondary outcomes include postoperative analgesic outcomes, which is NRS score at rest and during activity, recorded at 12, 24, 36, 48, and 72 h post‐surgery; postoperative knee function, which is the degree of movement defined by the range of motion (ROM); postoperative inflammatory response, including the level of HS‐CRP in blood and the level of TNF‐α and IL‐1β in joint fluid; the incidence of postoperative nausea and vomiting (PONV) and other postoperative complications (infection, deep vein thrombosis of the lower extremities, neurological complications). ROM is the maximum angle of active movement measured using a medical orthopedic goniometer. The medical orthopedic goniometer is positioned along the longitudinal axes of the tibia and femur, and the angle between them represents the ROM.

### Sample Size and Statistical Analysis

There is no specific data available regarding the NRS scores for a 6‐meter walk test undergoing knee arthroplasty with either perineural or intravenous betamethasone. Based on our pilot study with US‐guided FNB, we observed a mean NRS score of 1.7 with a standard deviation of 0.7 at a 6‐meter walk test in the NS group, a mean NRS score of 1.2 with a standard deviation of 0.6 in the PNB group, and a mean NRS score of 1.3 with a standard deviation of 0.5 in the IVB group. Given α = 0.05 and a power of 90%, we estimated that 46 individuals would be required in each group to test our hypothesis by one‐way analysis of variance (ANOVA) assuming equal variances (F‐Tests). Fifty‐three patients were included in each group to account for block failure, incomplete data, and loss of follow‐up.

The data are presented as mean ± standard deviation, median (IQR), or number (proportion), as appropriate. Statistical analysis was conducted using IBM SPSS Statistics V.26 (Phoenix, AZ, USA). The normality of continuous variables was tested using the Shapiro–Wilk test, followed by either one‐way ANOVA or the Kruskal‐Wallis rank sum test for comparison. For categorical variables, either the Pearson χ^2^ test or Fisher's exact test was employed for comparison. *p* < 0.05 was considered statistically significant.

## Results

**FIGURE 1:**
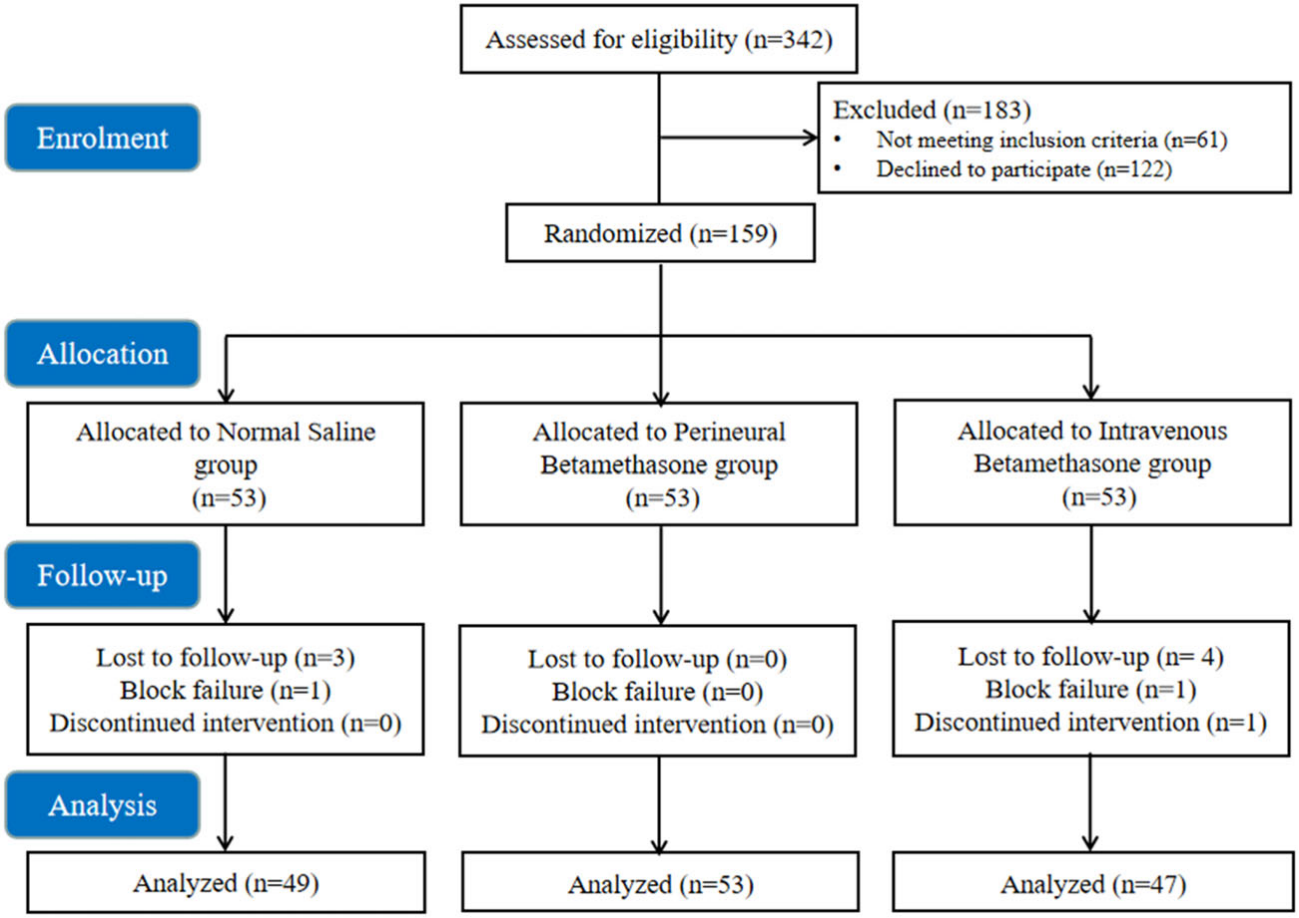
The Consolidated Standards of Reporting Trials (CONSORT) flow diagram.

Figure 1 shows the Consolidated Standards of Reporting Trials (CONSORT) flow diagram, which illustrates the study's conduct in detail. From June 2022 and February 2023, a total of 342 patients were evaluated. Among them, 61 patients either did not meet the inclusion criteria or met the exclusion criteria, while 122 declined to participate in the study. Ultimately, 159 patients were randomized, and 149 patients completed the study protocol and were included in the final analysis. The baseline characteristics of the included patients are shown in Table 1.

**TABLE 1:** Characteristics of patients with FNB for knee arthroplasty.

	NS group (*n* = 49)	PNB group (*n* = 53)	IVB group (*n* = 47)	*p* value
Age (years)	65.4 ± 6.8 [51, 79]	65.7 ± 6.1 [53, 79]	65.7 ± 6.6 [54, 80]	0.950
Sex				0.611
Male	11 (22.4%)	8 (15.1%)	8 (17%)	
Female	38 (77.6%)	45 (84.9%)	39 (83%)	
BMI (kg/m^2^)	26.4 ± 2.9 [20.8, 32.2]	27.0 ± 3.3 [20.8, 35.7]	27.9 ± 3.1 [20.7, 39.1]	0.083
ASA physical status				0.374
I	4 (8.2%)	2 (3.8%)	1 (4.7%)	
II	45 (91.8%)	51 (96.2%)	45 (94.6%)	
III	0 (0%)	0 (0%)	1 (0.7%)	
Duration of surgery (min)	99.5 ± 22.0 [60, 145]	91.2 ± 20.5 [65, 145]	93.2 ± 21.9 [60, 150]	0.135
Comorbidities				
Hypertension	24 (48.9%)	19 (35.4%)	27 (57.4%)	0.092
CHD	12 (24.4%)	9 (17.0%)	6 (12.8%)	0.318
Cerebral infarction	3 (6.1%)	1 (1.8%)	2 (4.3%)	0.551
Other diseases	5 (10.2%)	8 (15.1%)	4 (8.5%)	0.556

*Note*: Values are presented as mean ± SD, [minimum, maximum], or n (percent).

Abbreviations: ASA, American Society of Anesthesiologists; BMI, Body Mass Index; CHD, coronary heart disease; IVB, intravenous betamethasone; NS, normal saline; PNB, perineural betamethasone.

### Analgesia

**FIGURE 2:**
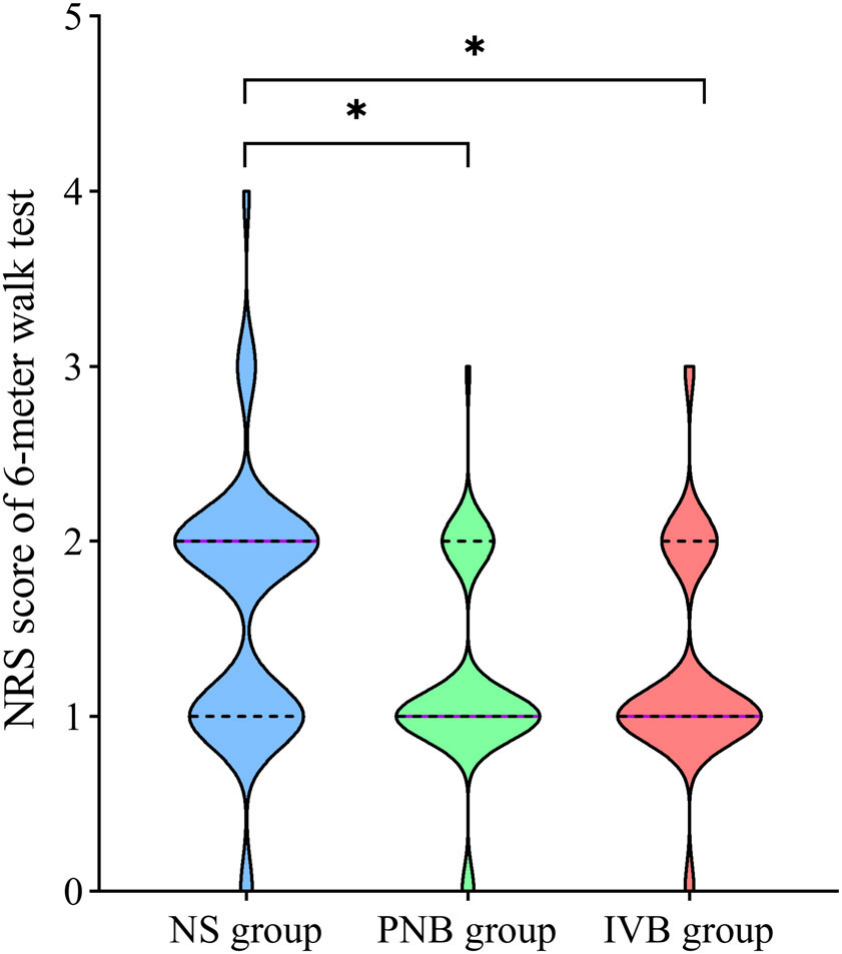
Violin plot of 6‐meter walk test numeric rating scale (NRS) score at 24 h after surgery. The purple solid line represents the median and the black dotted line represents interquartile range (IQR). *Statistically significant difference between groups (Kruskal–Wallis test), *p* < 0.05.

In the 6‐meter walk test 24 h after the operation, the median (IQR) NRS scores for the NS group, PNB group, and IVB group were 2.0 (1.0–2.0), 1.0 (1.0–2.0), 1.0 (1.0–2.0), respectively. Compared with the NS group, the median NRS score was significantly lower in the PNB group and IVB group (*p* = 0.003, Figure 2). However, there was no statistically significant difference between the PNB group and IVB group (*p* = 1.000). The mean difference observed in the 6‐meter walk test reached the clinically significant threshold, as indicated by the MCID value. The median NRS score for the 6‐meter walk test at 48 h showed results similar to those at 24 h (Table 2).

**TABLE 2:** Postoperative outcomes of patients with FNB for knee arthroplasty.

	NS group (*n* = 49)	PNB group (*n* = 53)	IVB group (*n* = 47)	*p* value^a^, ^b^
NRS score of a 6‐meter walk test				
P 24 h	2.0 (1.0–2.0)^c^, ^d^	1.0 (1.0–2.0)	1.0 (1.0–2.0)	0.003
P 48 h	2.0 (2.0–3.0)^c^, ^d^	1.0 (1.0–2.0)	1.0 (1.0–2.0)	0.006
Pain NRS score				
At rest				
P 12 h	0.0 (0.0–0.0)	0.0 (0.0–0.0)	0.0 (0.0–0.0)	0.360
P 24 h	0.0 (0.0–0.0)	0.0 (0.0–0.0)	0.0 (0.0–0.0)	0.719
P 36 h	1.0 (1.0–2.0)	1.0 (0.5–1.0)	1.0 (1.0–1.0)	0.655
P 48 h	1.0 (0.0–1.5)	1.0 (1.0–1.0)	1.0 (1.0–1.0)	0.828
P 72h	1.0 (1.0–2.0)	1.0 (1.0–2.0)	1.0 (1.0–1.0)	0.875
At 90° flexion				
P 24 h	1.0 (1.0–2.0)^c^	1.0 (0.0–2.0)	1.0 (0.0–2.0)	0.031
P 36 h	1.0 (1.0–2.0)	1.0 (1.0–2.0)	1.0 (1.0–2.0)	0.132
P 48 h	2.0 (1.0–2.0)	1.0 (1.0–2.0)	1.0 (1.0–2.0)	0.363
P 72 h	1.0 (1.0–2.0)	1.0 (1.0–2.0)	1.0 (1.0–2.0)	0.515
At 100° flexion				
P 24 h	2.0 (2.0–3.0)	2.0 (1.5–3.0)	2.0 (2.0–3.0)	0.235
P 36 h	3.0 (2.0–3.0)^c^	2.0 (2.0–3.0)	2.0 (2.0–3.0)	0.013
P 48 h	2.0 (2.0–3.0)	2.0 (2.0–3.0)	2.0 (2.0–3.0)	0.711
P 72 h	2.0 (2.0–3.0)	1.0 (2.0–3.0)	2.0 (2.0–3.0)	0.655
At 110° flexion				
P 24 h	3.0 (2.0–4.0)	3.0 (2.0–4.0)	3.0 (2.0–4.0)	0.471
P 36 h	4.0 (3.0–4.0)^c^	3.0 (2.0–4.0)	4.0 (2.0–4.0)	0.028
P 48 h	4.0 (3.0–4.0)	4.0 (2.0–4.0)	3.0 (2.0–4.0)	0.525
P 72 h	4.0 (3.0–4.3)	3.0 (2.0–5.0)	4.0 (3.0–4.0)	0.453
At 120° flexion				
P 36 h	4.0 (4.0–6.0)	4.0 (3.0–6.0)	4.0 (4.0–4.0)	0.134
P 48 h	4.0 (4.0–6.0)	4.0 (3.5–6.0)	4.0 (3.0–4.0)	0.269
P 72 h	4.0 (4.0–5.5)	4.0 (3.5–6.0)	4.0 (4.0–5.0)	0.701
ROM				
P 24 h	90 (90–90)^c^	90 (90–100)	90 (90–100)	0.003
P 36 h	90 (80–90)^c^	90 (90–100)	90 (90–100)	0.048
P 48 h	90 (90–100)^c^, ^d^	100 (90–100)	100 (90–100)	0.010
P 72 h	100 (90–107.5)	100 (97.5–110)	100 (100–110)	0.201

*Note*: Data are presented as mean (SD) or median (IQR).

Abbreviations: IVB, intravenous betamethasone; NRS, numeric rating scale; NS, normal saline; PNB, perineural betamethasone; ROM, Range of motion.

^a^ Kruskal‐Wallis test of any difference across the 3 study groups.

^b^ *P* values were adjusted by Bonferroni correction for multiple tests.

^c^ There is a difference between the NS group and the PVB group.

^d^ There is a difference between the NS group and IVB group.

Postoperative NRS scores at rest did not show a significant difference among the NS group, PNB group, and IVB group. However, at 24 h post‐surgery, the NRS score showed a significant difference between the PNB group and the NS group at 90° knee flexion, while no statistical difference was observed between the PNB group and the IVB group. The median NRS score with 100° knee flexion was lower in both the PNB group and IVB group compared to the NS group at 36 h. At 36 h post‐surgery, the median NRS scores for patients in the NS group, PNB group, and IVB group were 4.0 (3.0–4.0), 3.0 (2.0–4.0), and 4.0 (2.0–4.0) respectively, when the knee was flexed to 110°. A statistically significant difference in NRS score was observed between the IVB and NS groups (*p* = 0.023), but no statistical difference was noted between the IVB and PNB groups. There was no significant statistical difference in the median NRS score of patients at 48 h and 72 h post‐operation.

### Inflammatory Response

**FIGURE 3:**
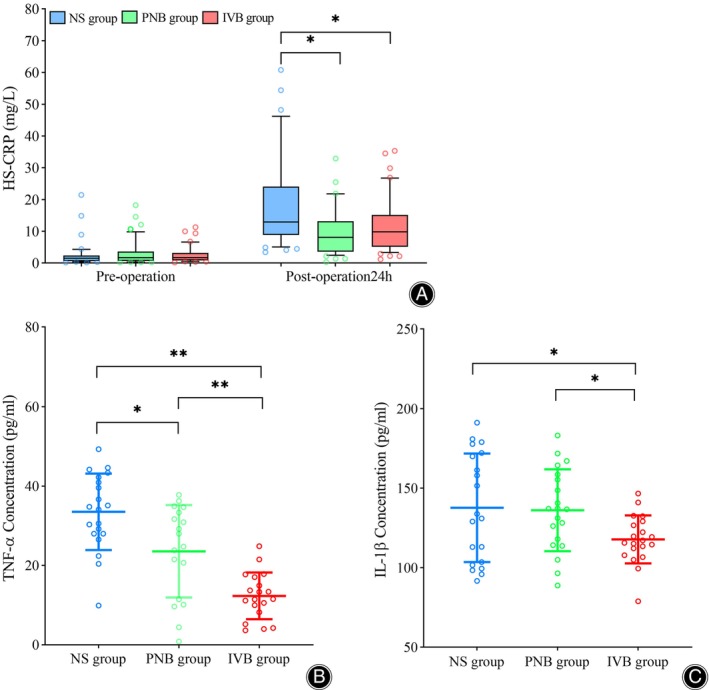
Inflammatory response. A: the concentration of HS‐CRP in blood at preoperative and postoperative. B: the concentration of TNF‐α in joint fluid at 24 h after surgery, C: the concentration of IL‐1β in joint fluid at 24 h after surgery. *Statistically significant difference, *p* < 0.05; **Statistically significant difference, *p* < 0.01.

The concentrations of TNF‐α were 33.5 ± 9.63, 23.6 ± 11.6, and 12.34 ± 5.9 pg./mL, the concentrations of IL‐1β were 137.7 ± 34.1, 136.2 ± 25.7, 117.9 ± 15.1 pg./mL, and the concentrations of HS‐CRP were 12.8 (8.8–25.6), 8.0 (4.1–13.1), 9.8 (5.3–15.0) mg/L for the NS, PNB and IIVB groups respectively (Table 3). As shown in Figure 3, the levels of TNF‐α, an indicator of the inflammatory response, were significantly lower in the IVB and PNB groups compared to the NS group. Furthermore, both the PNB and IVB groups showed significant differences in the concentrations of IL‐1β and HS‐CRP compared to the NS group.

**TABLE 3:** Inflammatory response and adverse events.

	NS group	PNB group	IVB group	*p* value^a^
TNF‐α (pg/mL)	33.5 ± 9.63^b^, ^c^, ^d^	23.6 ± 11.6	12.34 ± 5.9	0.000
IL‐1β (pg/mL)	137.7 ± 34.1^c^, ^d^	136.2 ± 25.7	117.9 ± 15.1	0.035
CRP (mg/L)				
Pre‐operation	1.5 (0.6–2.6)	1.5 (0. 7–3.5)	1.7 (0.8–3.2)	0.655
Post‐operation	12.8 (8.8–25.6)^b^, ^c^	8.0 (4.1–13.1)	9.8 (5.3–15.0)	0.003
PONV^e^	9 (18.4%)	8 (15.1%)	7 (14.9%)	0.871
Urinary retention^e^	2 (4.1%)	3 (5.7%)	2 (4.3%)	0.918
Infection	0 (0.0%)	1 (1.9%)	1 (2.1%)	0.605
VTE	0 (0.0%)	0 (0.0%)	0 (0.0%)	1.000
Persistent nerve palsy	0 (0.0%)	0 (0.0%)	0 (0.0%)	1.000

*Notes*: Data are presented as mean (SD), median (IQR), or number (percentage).

Abbreviations: CRP, C‐reactive protein; IVB, intravenous betamethasone; NS, normal saline; PNB, perineural betamethasone; PONV, postoperative nausea or vomiting; VTE, venous thromboembolism.

^a^ *p* values are calculated with Pearson χ2 test.

^b^ There is a difference between the NS group and the PVB group.

^c^ There is a difference between the NS group and IVB group.

^d^ There is a difference between the PNB group and IVB group.

^e^ Incidence within 48 h after surgery.

### Knee Function

ROM of the knee showed a difference at 24 h post‐operation (NS group 90 [90–90] *versus* PNB group 90 [90–100] *versus* IVB group 90 [90–100], *p* = 0.003), at 36 h post‐operation (NS group 90 [80–90] *versus* PNB group 90 [90–100] *versus* IVB group 90 [90–100], *p* = 0.048), and 48 h post‐operation (NS group 90 [90–100] *versus* PNB group 100 [90–100] *versus* IVB group 100 [90–100], *p* = 0.010), but no difference was observed at 72 h post‐operation (NS group 100 [90–107.5] *versus* PNB group 100 [97.5–110] *versus* IVB group 100 [100–110], *p* = 0.201).

### Adverse Events

No significant difference was observed in the occurrence of adverse events, such as PONV, urinary tract infection, lower extremity deep vein thrombosis, and infection, among the NS group, PNB group, and IVB group (Table 3).

## Discussion

In this randomized, placebo‐controlled trial, we evaluated and compared the impact of FNB combined with betamethasone on postoperative pain relief, inflammatory response, and knee function following knee arthroplasty. Compared to the NS group, the PNB group and IVB group showed an enhancement in postoperative analgesia and a decrease in the levels of pro‐inflammatory factors such as IL‐1β and TNF‐α. The NRS scores for the 6‐meter walking test at 24 h and 48 h post‐operation were significantly lower in the PNB and IVB groups compared to the NS group, suggesting that administration of peripheral or intravenous betamethasone could been beneficial for early postoperative recovery and physical activity in patients.

Despite ERAS being extensively promoted in orthopedics, there remains a lack of consensus on the optimal protocol for facilitating early mobilization after surgery.^22^ In our study, there was a significant reduction in the median NRS scores during knee flexion activities at 90° in both IVB and PNB groups after 24 h of surgery. Furthermore, at 36 h post‐surgery, the NRS scores were notably lower in the PNB and IVB groups during knee flexion activity at 100° and 110°. Active knee flexion, as an indicator of early postoperative activities, could reduce the risk of deep vein thrombosis and avoid knee stiffness. In clinical practice, postoperative pain can hinder patients' postoperative activity, potentially leading to postoperative knee stiffness, which can, in turn, result in reluctance to engage in activity, thereby creating a self‐perpetuating circle. In the study conducted by Rahangdale *et al*.,^23^ it was observed that perineural or intravenous administration of 8 mg dexamethasone increased the duration of analgesia for foot and ankle surgery compared to normal saline. Consistent with previous studies, our results demonstrated perineural betamethasone and intravenous betamethasone extended the duration of analgesia.^14^, ^15^ Our study also demonstrated intravenous or perineural administration of betamethasone within 48 h after surgery improved knee function, which was consistent with a previous study by Keohane *et al*.^2^ Although Aliste *et al*.^15^ have reported that perineural dexamethasone administration led to an extended period of motor block, sensory block, and postoperative analgesia in axillary brachial plexus blocks as compared to intravenous 8 mg dexamethasone. Although the differences between the two approaches were statistically significant, they were not clinically significant.

### Highlighting the Advantages of Femoral Nerve Block over Adductor Canal Block

Recently, the popularity of FNB has declined due to the risk of falls and quadriceps weakness. As a result, an adductor canal block (ACB) has gained notable popularity in many medical practices.^24^ However, there is no conclusive evidence to establish the superiority of ACB in established ERAS pathways.^22^ Individuals suffering from knee osteoarthritis are mostly elderly patients with underlying medical conditions. The mean arterial pressure (MAP) during tourniquet application in FNB is lower than that in ACB, while the MAP is higher than that in ACB when the tourniquet is released,^25^ which suggests that FNB could provide more stable hemodynamics. Studies have shown that there is no difference in quadriceps strength at the 24‐hour mark between the FNB and ACB.^25^, ^26^ Therefore, FNB could better relief from the intraoperative tourniquet reaction without affecting quadriceps strength compared to ACB at 24 h.

### The Mechanisms of Betamethasone to Prolong Postoperative Analgesia

The mechanism responsible for the prolonged duration of postoperative analgesia with betamethasone is not yet fully understood. Some suggested pharmacological mechanisms of long‐acting glucocorticoids include systemic anti‐inflammatory effects, perineural vasoconstriction induced by glucocorticoids to delay local anesthetic absorption, and the direct effects of glucocorticoids on nerve conduction.^4^, ^27^, ^28^, ^29^ The anti‐inflammatory properties of glucocorticoids are considered one of the reasons for sustained analgesia after nerve blocks. Glucocorticoids, upon entering the cells, bind to and activate glucocorticoid receptors present in the cytoplasm. This leads to the binding of the receptor with the glucocorticoid response element (GRE) present in the nucleus, ultimately resulting in the inhibition of inflammatory gene transcription, and an increase in the transcription of anti‐inflammatory genes. As a result of this process, the production of anti‐inflammatory proteins such as lipocortin‐1 is increased while the production of inflammatory proteins such as IL‐1β, TNF‐α, COX‐2, and endothelin is decreased.^16^, ^27^, ^30^ This study has shown that both perineural and intravenous administration of betamethasone reduced the levels of IL‐1β and TNF‐α in joint fluid, as well as HS‐CRP in blood. This supports the idea that the improved postoperative analgesic effect of betamethasone is related to its systemic anti‐inflammatory properties. Glucocorticoids are also believed to reduce ectopic neuronal firing and activate inhibitory potassium channels on C fibers, which in turn reduces the severity of pain.^14^, ^31^

### Evaluating the Perioperative Safety of Betamethasone

Studies have reported the perioperative safety of betamethasone and dexamethasone.^32^, ^33^, ^34^ In these studies, participants were randomly assigned to receive either dexamethasone or a placebo. The results indicated there was no significant difference in postoperative complication rate or mortality between the two groups. In a study conducted by Watanabe *et al*., it was shown that the addition of perineural betamethasone to ropivacaine in interscalene brachial plexus did not result in any adverse events.^19^ This is consistent with our study that there were no significant differences in the incidence of surgical site infection, urinary tract infection, lower extremity deep vein thrombosis, and nerve injury among patients. These studies provide evidence to support the idea that long‐acting glucocorticoids such as betamethasone can enhance the quality of the block and extend the duration of the analgesia. Previous research has demonstrated the efficacy of intravenous injection of glucocorticoids in preventing PONV.^35^ Kim *et al*. reported no significant difference in the incidence of nausea and vomiting after sciatic nerve block between intravenous dexamethasone 2.5, 5, 10 mg, and placebo,^13^ which is consistent with our results. It may be associated with the administration of oral ondansetron to the patients in our study.

The safety of perineural administration of betamethasone and dexamethasone has been a subject of debate. It is important to note that the use of perineural betamethasone is considered off‐label. Animal experiments have demonstrated that combining ropivacaine with dexamethasone resulted in a significant increase in neurotoxicity as the concentration of dexamethasone increased from 66 μg/mL to 133 μg/mL.^36^ However, it is important to acknowledge that the study has significant confounding factors that could affect the accuracy of the results. The observed neurotoxicity may be related to the presence of preservatives like polyethylene glycol or insoluble steroid particles.^37^ Recent research has also suggested a neuroprotective effect of dexamethasone by reducing nerve cell injury induced by bupivacaine and lidocaine.^37^ In addition, the possibility of perineural application of betamethasone may also be supported by the prior extensive use of glucocorticoids for radicular pain.^38^, ^39^ Thus, considering the effectiveness and theoretical safety concerns, we recommend that intravenous betamethasone should be preferred for patients undergoing regional analgesia in postoperative pain management.

### Strengths and Limitations

This study innovatively assesses whether administering betamethasone through different pathways enhances early mobility and postoperative recovery in the context of a multimodal analgesic regimen. Another strength of this trial lies in its meticulous methodological approach, employing rigorous techniques such as blinding, allocation concealment, and intention‐to‐treat analysis to minimize bias. There are several limitations to our study. First, this study only followed up the complications related to betamethasone neurotoxicity for 1 month. Further exploration and long‐term follow‐up are needed to understand the concentration and time‐dependence nature of betamethasone's neurotoxicity when combined with ropivacaine. Second, for inflammation response research, we only randomly selected joint fluids samples from 10 patients in each group and tested indicators such as TNF‐α and IL‐1β, rather than examining the entire population.

## Conclusion

In conclusion, the utilization of perineural or intravenous betamethasone with US‐guided FNB during knee arthroplasty results in better analgesia, which facilitates early postoperative activity and recovery. Nevertheless, it is worth noting that the postoperative analgesic effects of perineural and intravenous betamethasone are comparable.

## Conflict of Interest Statement

The authors declare that they have no conflict of interest.

## Author Contributions

Binglong Li mainly completed the study design and conduct, data collection and analysis, and a draft of the manuscript. Xiaomei Yang completed a study design and conducted, data collection and analysis, and revision of the manuscript. Fang Liu completed the execution of the trial, data collection and analysis. Huang Huang completed revision of the manuscript. Baoqing Zhang, Xuezhou Li and Qunshan Lu completed data collection and analysis. Peilai Liu and Lixia Fan completed the study concept, study design, and revision of the manuscript. All authors read and approved the final manuscript.

## Funding

This work was supported by the Shandong Provincial Natural Science Foundation of China (ZR2022MH052), China Postdoctoral Science Foundation (No. 2021M691944), and Special funds for comfortable medical anesthesia optimization of Shandong Provincial Medical Association (YXH2021ZX015) to Xiaomei Yang, and the Horizontal Project of Shandong University (12671818) to Peilai Liu.

## Supporting information


**TABLE S1.** Diagnostic criteria of knee osteoarthritis.
