# Supplementary material for: Effect of Perineural or Intravenous Betamethasone on Femoral Nerve Block Outcomes in Knee Arthroplasty: A Randomized, Controlled Study
Source: Orthop Surg. 2024 Feb 21;16(4):873–81. doi: 10.1111/os.14018 (PMC10984829; doi:10.1111/os.14018)
Supplement: Supplementary file 1 — TABLE S1. Diagnostic criteria of knee osteoarthritis. [file OS-16-873-s001.docx]

**Supplementary Table**

|  | Diagnostic criteria of knee osteoarthritis |
| --- | --- |
| 1 | Recurrent knee pain over the past month |
| 2 | A sensation of bone rubbing during activity |
| 3 | Morning stiffness lasts ≤30 min |
| 4 | Age ≥40 years old |
| 5 | X-ray (standing weight-bearing): narrow of the joint space, formation of marginal osteophytes and/or subchondral sclerosis |
| 6 | X-ray Kellgren & Lawrence grade ≥ II |

Supplementary Table 1 Diagnostic criteria of knee osteoarthritis
